# Double‐Gyroid Nanostructure Formation by Aggregation‐Induced Atropisomerization and Co‐Assembly of Ionic Liquid‐Crystalline Amphiphiles

**DOI:** 10.1002/anie.202000424

**Published:** 2020-03-17

**Authors:** Nanami Uemura, Tsubasa Kobayashi, Shintaro Yoshida, Ya‐xin Li, Karel Goossens, Xiangbing Zeng, Go Watanabe, Takahiro Ichikawa

**Affiliations:** ^1^ Department of Biotechnology Tokyo University of Agriculture and Technology Tokyo 184-8588 Japan; ^2^ Department of Physics School of Science Kitasato University Sagamihara Kanagawa 252-0373 Japan; ^3^ Department of Materials Science and Engineering University of Sheffield Sheffield S1 3JD UK; ^4^ Center for Multidimensional Carbon Materials (CMCM) Institute for Basic Science (IBS) Ulsan 44919 Republic of Korea

**Keywords:** atropisomers, bicontinuous cubic phase, double-gyroid structures, liquid crystals, self-organization

## Abstract

We report a new molecular‐design principle for creating double‐gyroid nanostructured molecular assemblies based on atropisomerization. Ionic amphiphiles containing two imidazolium rings close to each other were designed and synthesized. NMR data revealed that the rotation of the imidazolium rings is restricted, with an activation energy as high as 63 kJ mol^−1^ in DMSO‐d_6_ solution (DFT prediction for a model compound in the vacuum: 90–100 kJ mol^−1^). Due to the restricted rotation, the amphiphiles feature “double” atropisomeric axes in their ionic segments and form three stable atropisomers: *meso*, *R*, and *S*. These isomers co‐organize into $Ia\bar{3}d$ ‐type bicontinuous cubic liquid‐crystalline mesophases through nanosegregation of the ionic and non‐ionic parts. Considering the intrinsic characteristic of $Ia\bar{3}d$ ‐type bicontinuous cubic structures that they are composed of intertwined right‐ and left‐handed single gyroids, we propose that the simultaneous presence of both *R*‐ and *S*‐atropisomers is an important contributor to the formation of double‐gyroid structures.

## Graphical abstract

**With both hands**: A new molecular‐design principle for creating double‐gyroid nanostructured molecular assemblies based on atropisomerization is reported. Ionic amphiphiles with rotationally restricted imidazolium rings are used. Three stable atropisomers co‐organize into bicontinuous cubic liquid‐crystalline mesophases through nanosegregation of the ionic and non‐ionic parts.

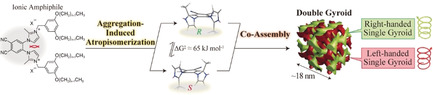

Double‐gyroid structures are a class of 3D periodic structures with an $Ia\bar{3}d$ cubic symmetry.^1^ They are composed of two interwoven networks of 3D branched channel structures. Owing to the unique structural characteristics, functional materials forming such bicontinuous cubic (Cub_bi_) structures have been actively pursued to achieve, for example, efficient mass transport,^2^ drug delivery,^3^ or catalysis.^4^ Several liquid‐crystalline (LC) molecules that adopt thermotropic Cub_bi_ mesophases have been shown to self‐organize into double‐gyroid nanostructures.^5^ This class of nanostructured materials has attracted increasing attention because of their potential utility as “alignment‐free” nanochanneled materials for size‐selective separation membranes^6^ or conductive matrices.^7^

Out of tens of thousands of reported LC molecules, relatively few exhibit Cub_bi_ phases.^5^, ^8^ The molecular requirements to achieve such mesophases are not yet fully understood, although there is a small degree of understanding on the characteristics of the molecular structures forming Cub_bi_ phases.^5^, ^6^, ^7^, ^8^, ^9^, ^10^ Because of the technological potential as nanochannel materials, it is important to establish molecular‐design principles for obtaining Cub_bi_ liquid crystals. One significant clue is the fact that double‐gyroid structures are composed of two screw helices of opposite handedness which are mirror images of each other.

Previous studies on thermotropic Cub_bi_ liquid crystals have identified roughly three mechanisms that may lead to $Ia\bar{3}d$ ‐type Cub_bi_ mesophases (Figure 1). In case I, achiral rod‐ or disc‐shaped LC molecules incidentally form, through nucleation, a left‐ or right‐handed helical assembly in one domain and a helix of opposite handedness in a neighboring domain to optimize space‐filling.5b, 11 Case II has been found for racemic mixtures of chiral LC molecules.^12^ When such mixtures form supramolecular assemblies, there is a possibility that, locally, the ratio of *R*‐ and *S*‐isomers slightly deviates from 1:1. Such deviations result in the formation of both right‐ and left‐handed domains and the creation of double gyroid structures. Case III was reported by Tschierske and co‐workers for polycatenar rod‐like molecules based on a 5,5′‐diphenylbithiophene core.^13^ These achiral compounds locally form right‐ or left‐handed chiral assemblies as a result of chirality synchronization of the close‐packed molecules. In this case, the enantiomerization has an activation energy of only 2.7 kJ mol^−1^. In all three cases, the LC molecules form twisted assemblies to reduce packing frustrations, and the supramolecular assemblies further organize into double‐gyroid structures.

**Figure 1:**
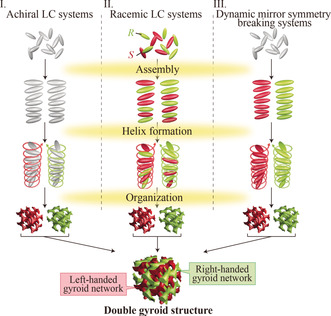
Schematic illustration of the different formation mechanisms for thermotropic $Ia\bar{3}d$ Cub_bi_ mesophases. Two helical nanostructures of opposite handedness co‐organize into double gyroid structures. Case I: Achiral molecules form neighboring right‐handed and left‐handed helix domains. Case II: Racemic mixtures of chiral molecules form two helix domains of opposite handedness. Case III: Dynamic mirror‐symmetry breaking of achiral molecules spontaneously produces right‐ and left‐handed helices.

Considering the importance of right‐ and left‐handed helix formation for inducing Cub_bi_ phases, we decided to focus on molecular building blocks that form atropisomers, that is, stereoisomers that may occur when free rotation about a particular bond is prohibited by, for example, steric hindrance.^14^ Atropisomers have been intensively explored for designing asymmetric catalysts.^15^ More recently, there have been several reports of organic compounds that form atropisomers only in aggregated states.^16^ One of the key factors that play a role in this phenomenon is the restriction of intramolecular rotation upon close‐packing of the molecules. We envisioned that exploitation of such aggregation‐induced atropisomerization (AIA) could be a new way of designing Cub_bi_ liquid crystals. The polycatenar rod‐like LCs that were reported by Tschierske and co‐workers^13^ can be considered to be the first examples of Cub_bi_ liquid crystals based on AIA phenomena.

In the present study, the molecular design was inspired by work of Claramunt et al., who synthesized benzene derivatives containing two 2‐methyl‐1‐imidazolyl groups.^17^ They reported that, for the *ortho*‐disubstituted derivatives, rotation about the C−N bonds between the benzene ring and the two imidazole rings (C_benzene_−N) is sterically hindered, giving rise to the formation of atropisomers. We modified the original structures of Claramunt et al. and designed a 1,2‐dicyanobenzene having two 2‐methyl‐1‐imidazolyl moieties in the 4‐ and 5‐positions (DCB‐Im_2_, Figure 2 a). To estimate the energy barrier for rotation of the imidazole rings, DFT calculations were performed for DCB‐Im_2_ in vacuum. After defining the dihedral angles *φ* and *ψ* as shown in Figure 2 b, we estimated the energy barriers for rotation about the C_benzene_−N bonds and constructed a 2D potential‐energy map (Figure 2 c). The map revealed four zones (I, I′, II, and III; indicated in blue) corresponding to stable conformers in which the two imidazole rings are tilted by circa 30° relative to the plane of the dicyanobenzene ring to form either “parallel” (*meso*) or “antiparallel” (*R* or *S*) isomers (Figure 2 d).^18^ It is noteworthy that the energy barriers that separate the four blue regions in Figure 2 c are about 90–100 kJ mol^−1^. Considering previous insights into the relationship between the rotation energy barrier and atropisomer formation,^19^ an energy barrier of circa 100 kJ mol^−1^ seems to be sufficient for inducing permanent atropisomers even in solution. It can be expected that a slightly smaller energy barrier is already effective for suppressing the interconversion of isomers in bulk aggregation states.

**Figure 2:**
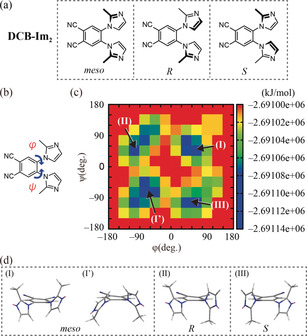
a) Molecular structure of DCB‐Im_2_. The molecule can adopt three stable conformations: *meso*, *R*, and *S*. b) Definition of dihedral angles *φ* and *ψ*. c), d) 2D potential energy map showing the energy barriers for rotation about the C_benzene_−N bonds (dihedral angles *φ* and *ψ*) in DCB‐Im_2_, as obtained from DFT calculations. Four stable conformer regions were identified and designated as I/I′, II, and III, corresponding to the *meso*, *R*, and *S* conformers, respectively.

Following twofold quaternization, the DCB‐Im_2_ fragment was used as the ionic headgroup of amphiphilic block molecules (**1**_*n*_‐X, Figure 3; *n*: number of carbon atoms in the alkyl chains, X: anion species) for which we examined the relationships between atropisomerization and self‐organization.

**Figure 3:**
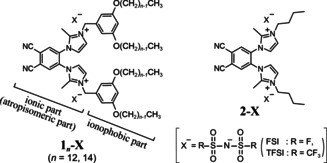
Molecular structures of ionic amphiphilic molecules **1**_*n*_‐X and model compounds **2**‐X.

To experimentally confirm the occurrence of atropisomerism for the ionic headgroup of **1**_*n*_‐X, a solution of compound **1**_14_‐TFSI in DMSO‐*d*_6_ was subjected to ^1^H NMR measurements at room temperature. Signals of the protons at the 4‐ and 5‐positions of the imidazolium rings (that is, H_4_ and H_5_) were observed as broad, overlapping peaks in the range 7.40–8.00 ppm. Through deconvolution of the overlapping peaks by Lorentzian peak‐fitting, we found that there were three peaks, a(7.75 ppm), b(7.66 ppm), and c(7.56 ppm) with a peak‐area ratio of about 3:5:2. To identify the three peaks, a NOESY experiment, which is a 2D NMR method to yield through‐space correlations via spin‐lattice relaxation, was performed. The obtained profile is shown in Figure 4. It should be noted that the proton signals of the methylene groups in the benzyl groups appear at 5.33 ppm (peak f) and those of the benzene rings appear at 6.34 and 6.46 ppm (peaks e and d), respectively. Since compound **1**_14_‐TFSI has two imidazolium rings, we define them as Im_1_ and Im_2_ as shown in Figure 4 a in order to make discussion clear. It is notable that peak b shows cross‐peaks with peak e and f. Taking into account the results of previous studies on NOESY experiments for imidazolium compounds,^20^ peak b could be assigned to H_4_, which is close to the benzyl group. Consequently, peak a and c can be assigned to H_5_ with consideration of the peak‐area ratio of the three peaks. Focusing on peak a and c, it can be seen that peak a shows weak correlations with peak e and f while peak c shows no correlation. Based on the previous studies referred to above,^20^ it becomes evident that the proton at 5‐position of the imidazolium ring shows no cross‐peak with the proton of the methylene groups at the 3‐position of the imidazolium ring. These insights lead us to conclude that the weak cross‐peak between peaks a and e,f are attributed to the correlation between H_5_ of Im_1_ and the protons of the benzyl group from the other imidazolium ring Im_2_. Since it is expected that H_5_ of Im_1_ gets closer to the methylene group of Im_2_ in the *R*/*S* conformers than in the *meso* conformer, peak a can be assigned to H_5_ in the *R*/*S* conformers while peak c can be assigned to the same proton in the *meso* conformer.

**Figure 4:**
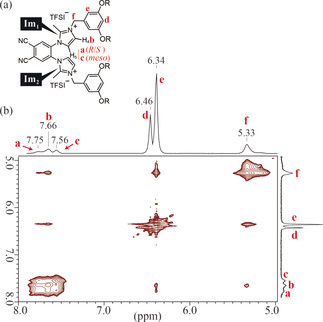
a) Molecular structure of **1**_14_‐TFSI. The two imidazolium rings are defined as Im_1_ and Im_2_. b) ^1^H NOESY NMR profile of **1**_14_‐TFSI in DMSO‐*d*_6_, recorded on a 400 MHz spectrometer at 23 °C.

With the aim to examine the temperature dependence of the atropisomerism, ^1^H NMR measurements were performed for a solution of compound **1**_14_‐TFSI in DMSO‐*d*_6_ at various temperatures (Figure 5). As mentioned above, H_4_ appears as a broad peak at 7.70 ppm (peak b) whereas H_5_ appears as two broad signals centered at slightly different ppm values depending on the type of conformer: 7.79 ppm for *R*/*S*‐conformers (peak a) and 7.60 ppm for *meso* conformers (peak c) at 30 °C. The peak‐area ratio between a(*R*/*S*) and c(*meso*) was measured to be 13:12, reflecting the relative populations of the isomers. Upon heating the solution, the two signals corresponding to the *R*/*S* and *meso* conformers gradually coalesced to become a single peak centered at 7.65 ppm. The latter value is consistent with the weighted average position of a(*R*/*S*) and c(*meso*). The observed behavior can be explained by the increasingly fast interconversion of the conformers upon heating. Based on the variable‐temperature ^1^H NMR data and using the Eyring equation,^19^ the energy barrier for rotation about the C_benzene_−N bonds was estimated to be 63 kJ mol^−1^ (see the Supporting Information). Similar experiments were carried out for model compound **2**‐TFSI. As for **1**_14_‐TFSI, the occurrence of *R*/*S* and *meso* isomers was observed for **2**‐TFSI, and the energy barrier for conformational interconversion was estimated to be 64 kJ mol^−1^ (see the Supporting Information, Figure S20). Although the aforementioned energy barriers are lower than the value of 100 kJ mol^−1^, which is considered to be a prerequisite for the permanent existence of different atropisomers, the results strongly suggest that, in solution, the rotation about the C_benzene_−N bonds is hindered to some extent. It can be expected that in bulk LC states, the rotation is even more restricted, giving rise to AIA phenomena. Further details about the NMR experiments can be found in the Supporting Information (Figures S21–S25).

**Figure 5:**
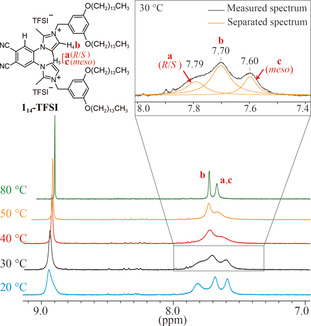
^1^H NMR spectra of **1**_14_‐TFSI in DMSO‐*d*_6_, recorded on a 500 MHz spectrometer over a range of temperatures (20–80 °C). Deconvolution of peaks in the range 7.40–8.00 ppm was achieved by Lorentzian peak‐fitting.

Next, the thermal phase behaviors of salts **1**_*n*_‐X in the bulk were examined using polarized‐light optical microscopy (POM), differential scanning calorimetry (DSC), and X‐ray diffraction measurements. Compounds **1**_*n*_‐X show thermotropic Cub_bi_ and hexagonal‐columnar (Col_h_) phases as a result of nanosegregation of the ionic moieties and the flexible, ionophobic molecular segments. The phase‐transition temperatures are summarized in Figure 6 a. Cub_bi_ phases were observed for **1**_14_‐FSI and **1**_14_‐TFSI. For example, upon cooling a sample of **1**_14_‐TFSI from its isotropic liquid (Iso) state, a focal conic texture appeared at 127 °C (Figure 6 b, left). Upon further cooling, the POM texture started to disappear at around 78 °C, indicating the transition to an optically isotropic Cub_bi_ phase (Figure 6 b, center and right).

**Figure 6:**
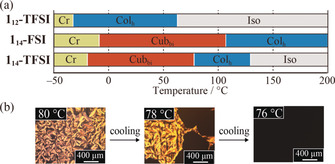
a) Thermotropic LC properties of **1**_*n*_‐X upon cooling. Cr, crystalline; Col_h_, hexagonal columnar; Cub_bi_, bicontinuous cubic; Iso, isotropic. c) Polarized‐light optical microscopy images of **1**_14_‐TFSI, showing the phase transition from the Col_h_ to Cub_bi_ phase.

Synchrotron‐based small‐angle X‐ray scattering (SAXS) measurements were performed to obtain further insight into the LC mesophases adopted by compounds **1**_14_‐FSI and **1**_14_‐TFSI. Figure 7 a shows SAXS patterns of **1**_14_‐FSI that were recorded at various temperatures during heating. The SAXS pattern recorded at 170 °C shows one intense peak and four weak peaks, which were indexed as the (10), (11), (20), (21), and (30) reflections of a 2D hexagonal arrangement of columns (hence, Col_h_) with a lattice parameter *a* of 40.6 Å. The SAXS pattern recorded at 120 °C shows two intense peaks and thirteen weak peaks, which were indexed as the (211), (220), (321), (400), (420), (332), (422), (521), (440), (611)/(532), (541), (631), (444), (543), and (633) reflections of a 3D cubic structure with $Ia\bar{3}d$ symmetry and a lattice parameter *a* of 89.8 Å. Further details are provided in the Supporting Information (Figures S17–S19). Based on the experimental SAXS intensities for the Col_h_ and Cub_bi_ phases, electron‐density maps were reconstructed (Figure 7 b). The high‐ and low‐electron‐density regions are colored in purple and red, respectively. The high‐density regions appear in the center of the nanochannels in the Col_h_ and Cub_bi_ phases. These results are consistent with a localization of the ionic parts of **1**_*n*_‐X in the interior of the nanochannels, as was previously found for wedge‐shaped ionic liquid crystals forming Col_h_ and Cub_bi_ phases.2b, 7a

**Figure 7:**
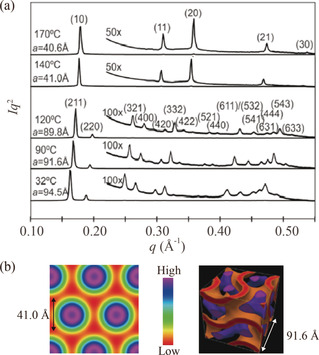
a) Small‐angle X‐ray scattering patterns of **1**_14_‐FSI, recorded at various temperatures during heating. b) Reconstructed electron density maps of the Col_h_ phase of **1**_14_‐FSI at 140 °C (2D map) and of the $Ia\bar{3}d$ ‐Cub_bi_ phase of **1**_14_‐FSI at 90 °C (3D map).

Based on the SAXS data and considering the molecular structures and volumes of compounds **1**_*n*_‐X, the organization into Col_h_ and Cub_bi_ mesophases occurs via self‐assembly of about three molecules into supramolecular discs that stack on top of each other (see Figure S26). As mentioned above, the NMR results in solution indicated that rotation about the C_benzene_−N_imidazolium_ bonds is restricted to some extent. The hindrance of free rotation is expected to significantly increase upon aggregation, thus endowing **1**_*n*_‐X with quasi‐permanent chirality as a result of AIA. We hypothesize that, upon formation of the bulk LC mesophases, packing frustrations are slightly reduced by locally enantiopure stacking of the *R*‐ and *S*‐enantiomers, respectively. Hence, the ratio of *R*‐ and *S*‐isomers may locally deviate from 1:1 (as shown in Figure 1, case II), leading to the creation of double‐gyroid structures. It should be noted that it is impossible to exclude the possibility that the present LC system proceeds via case III if **1**_*n*_‐X molecules have an ability to change the conformation of other neighboring molecules, as is the case for the LC systems reproted by Tschierske.^13^ It should also be noted that there are many double‐gyroid structures of non‐chiral compounds, such as block copolymers and dendrimers,1c, 2a, 21 which means that helical molecular assemblies or chiral molecular structures are not essential for the formation of double‐gyroid structures. For inducing the formation of double‐gyroid structures in the case of these materials, it is generally understood that the volume balance between the mutually incompatible blocks is known to be a key factor. This idea is also true in the case of LC block molecules. Considering a fact that only a limited number of ionic liquid crystals have been reported to exhibit thermotropic Cub_bi_ phases although a variety of ionic LC compounds have been designed,2b, 7, 22, 23 we believe that the emergence of double‐gyroid $Ia\bar{3}d$ cubic phases for **1**_14_‐X is also partially promoted by their organization into twisted assemblies as well as the effects of the volume balance between the ionic/non‐ionic parts.

In summary, we have designed novel ionic block molecules **1**_*n*_‐X that may adopt three stable conformations. NMR‐spectroscopic measurements revealed a significant energy barrier of ≈63 kJ mol^−1^ in DMSO‐*d*_6_ solution for the interconversion between the stable conformers of **1**_*n*_‐X through rotation about the C−N bonds linking the benzene and imidazolium rings. The high experimentally obtained energy barrier was consistent with DFT‐calculation results for a model compound. Compounds **1**_14_‐FSI and **1**_14_‐TFSI form thermotropic $Ia\bar{3}d$ ‐type bicontinuous cubic mesophases composed of two interdigitated single‐gyroid structures of opposite handedness. We propose that close‐packing of molecules **1**_14_‐X stabilizes the different atropisomers due to AIA, which, in turn, promotes the formation of helical structures with different senses. This concept constitutes a new molecular‐design principle, not only for developing bicontinuous cubic liquid crystals, but also to obtain functional small molecules and polymers that self‐organize into helical supramolecular assemblies.^24^, ^25^ For instance, the architecture of salts **1**_*n*_‐X can be used to design new chiral ionic liquids, which have attracted attention as media for asymmetric organic synthesis and chiral chromatography.^26^

## Conflict of interest

The authors declare no conflict of interest.
